# Roxadustat effectiveness versus ESAs in peritoneal dialysis patients during the COVID-19 pandemic: A retrospective study

**DOI:** 10.1371/journal.pone.0320536

**Published:** 2025-03-26

**Authors:** Jie Liu, Kefan Zhou, Chen Meng, Zhuzhu Liu, Ruihua Huang, Yousuf Waheed, Fan Yang, Kun Liu, Jiaqi Zhao, Lin Zhang, Xiaoyan Yu, Shuang Li, Tianyu Li, Yanshan Tong, Xiaodan Wei, Chuankuo Tian, Dong Sun, Xinglei Zhou

**Affiliations:** 1 Department of Nephrology, The Second Affiliated Hospital of Xuzhou Medical University, Xuzhou , China; 2 Kangda College of Nanjing Medical University, Lianyungang, China; 3 Department of Nephrology, Affiliated Hospital of Xuzhou Medical University, Xuzhou, China; 4 Department of Nephrology, Beijing Aerospace General Hospital, Beijing, China; 5 Department of Internal Medicine and Diagnostics, Xuzhou Medical University, Xuzhou, China; Taylor's University - Lakeside Campus: Taylor's University, MALAYSIA

## Abstract

**Background:**

The COVID-19 pandemic has made treating renal anemia in chronic kidney disease (CKD) patients undergoing peritoneal dialysis (PD) difficult. The current study aims to compare roxadustat with erythropoiesis-stimulating agents (ESAs) during the COVID-19 pandemic.

**Methods:**

We conducted a single-center, retrospective study during the COVID-19 outbreak in China, from December 7, 2022, to January 31, 2023. The study involved patients undergoing PD who were divided based on the medication used to treat renal anemia; the roxadustat group (n = 34) and the ESAs group (n = 120). We analyzed the effectiveness of treating anemia, cost, medication adherence, and clinical outcomes related to COVID-19. Patients were followed up for 9 months.

**Results:**

The baseline of hemoglobin levels was (110.03 ± 1.71 g/L in the roxadustat and 110.1 ± 1.52 g/L in the ESAs groups, respectively), after 9 months of inspections, the levels of hemoglobin were (121.26 ± 2.03 g/L in the roxadustat and 118.49 ± 1.35 g/L in the ESAs groups, respectively). The roxadustat subgroup analysis indicated that total cholesterol and low-density lipoprotein levels in the roxadustat group decreased from baseline in subjects not receiving statins (3.39 ± 0.12 vs. 4.2 ± 0.21 mmol/L and 2.21 ± 0.23 vs. 3.65 ± 0.37 mmol/L, *P < 0.05*). The Morisky score of the roxadustat group was higher [7 (5, 8) vs. 6 (4, 8), *P* < 0.01]. The drug cost of the roxadustat group was higher, but another additional cost for correcting anemia was significantly reduced. The infection rate of COVID-19 and the mortality rate caused by COVID-19 were lower in roxadustat group.

**Conclusion:**

During the COVID-19 pandemic, both roxadustat and ESAs effectively improved renal anemia in PD patients, however, the roxadustat group experienced less additional costs for anemia correction and better medication compliance.

## Introduction

The COVID-19 pandemic, which began in December 2019, has significantly impacted the global social economy and posed serious threats to public health worldwide. Compared with the general public, dialysis patients are more vulnerable to COVID-19 infection and have higher mortality rates [1,2]. One of the main reasons is that dialysis patients tend to have low resistance due to multiple complications, such as diabetes, hypertension (HTN), cardiovascular (CVD), cerebrovascular diseases, etc [3–5]. Additionally, these patients need regular hospital check-ups, which makes it challenging to maintain safe social distancing at all times.

Renal anemia is one of the common complications of dialysis patients, which not only affects the quality of life but also increases CVD, cerebrovascular, and mortality risks [6,7]. Previously, the mechanisms of renal anemia have been discussed; anemia in CKD patients is characterized by hyperproliferative and normocytic features [8]. PD is one of the kidney replacement therapies for patients with end-stage kidney disease (ESKD); it can mainly be operated by patients at home and can better protect residual renal function. PD patients account for about 11% of the global dialysis population [9]. Therefore, addressing the treatment of renal anemia in this population is significantly important.

Roxadustat and erythropoiesis-stimulating agents (ESAs) are commonly used in the treatment of renal anemia. Roxadustat is an oral medication for treating renal anemia; it is a hypoxia-inducible factor prolyl hydroxylase inhibitor (HIF-PHI). It increases endogenous erythropoietin concentration by inhibiting the HIF prolyl hydroxylase, as well as by mimicking the cellular response to hypoxia, thereby increasing the HIF activity [8]. ESAs need to be administered by injection, which acts directly on erythroid progenitor cells, thus playing the hematopoietic function [10,11]. Both drugs are effective in the treatment of renal anemia, but they also have some disadvantages. For example, injection administration of ESAs not only increases the patient’s pain but also increases the risk of infection and thus reduces the patient’s compliance [12]. Numerous clinical studies have confirmed the effectiveness of roxadustat in the treatment of renal anemia [13–15]. Its oral administration offers an advantage in lowering the risk of infection during the pandemic. However, concerns regarding its high medical cost and unknown effectiveness in the pandemic environment are still worthy of further discussion.

It is crucial to explore different ways to better ensure the medical safety of PD patients during a pandemic, such as COVID-19, particularly during the treatment of renal anemia. After reviewing existing literature, few clinical studies have addressed this area. There was only one self-controlled study on PD patients who were switched to roxadustat from ESAs due to the impact of COVID-19, which only included 29 patients and did not provide an inter-group comparison [16]. The objective of this study was to comprehensively and objectively compare the effectiveness, medication adherence, drug expenditure, and clinical outcomes of continuing to use roxadustat or ESAs in PD patients in the context of the COVID-19 pandemic, to provide a reference for the treatment of renal anemia during other pandemics.

## Methods

### Patients

**Fig 1:**
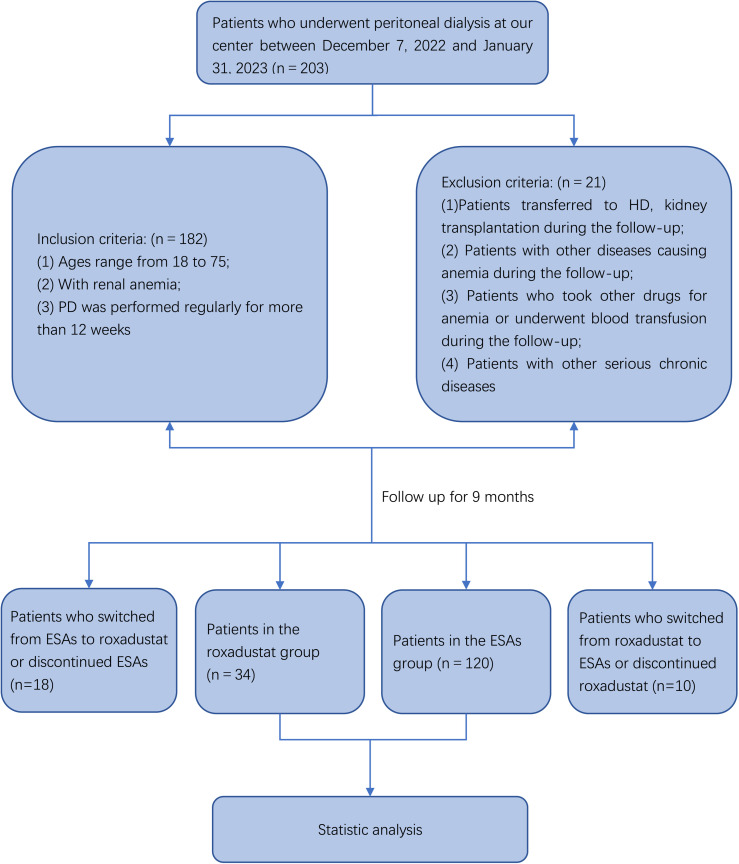
Patient flow diagram. PD: peritoneal dialysis; HD: hemodialysis; ESAs: erythropoiesis-stimulating agents.

We conducted a single-center, retrospective study on patients undergoing PD at our center during the local intensive outbreak of COVID-19, specifically from the time between December 7, 2022, to January 31, 2023, with a follow-up period of 9 months. Based on the different drugs used to treat renal anemia, we classified the patients into two groups, the roxadustat group (n = 34) and the ESAs group (n = 120). Importantly, neither group had any change to the medications for anemia three months before enrolment. Patients in both groups had adequate iron levels of ferritin (SF) >100 ng/ml, and transferrin saturation (TSAT) >20% at the initial stage of this study. The inclusion criteria were: (1) Patients aged 18–75 years; (2) Patients receiving PD regularly for more than 3 months. The exclusion criteria were: (1) Patients who transferred to hemodialysis (HD) or kidney transplantation during follow-up; (2) Patients who had other conditions that cause anemia; (3) Patients receiving other drugs to treat anemia; (4) Patients with other serious chronic diseases, such as malignant tumors, severe heart failure. The flow chart of our study is shown in Fig 1. The study was approved by the Medical Ethics Committee of the Second Affiliated Hospital of Xuzhou Medical University with approval number: [2023]090604, and also completed Chinese Clinical Trial Registration (registration number: ChiCTR2400086902). This study was exempted from obtaining informed consent due to its retrospective character.

### Study Design and Setting

This study only included only patients who underwent PD at our center, and the follow-up period was up to 9 months. We determined the initial dose of roxadustat treatment according to the drug instructions. Patients weighing 45–60 kg were given 100 mg three times a week orally. Patients weighing ≥ 60 kg were given 120 mg three times a week orally. We determine the initial dose of ESAs according to the instructions, the patient’s body weight, hemoglobin (Hb) concentration, and clinical conditions. Usually, the initial dose of ESAs should be 50~150 IU/kg per week, 1–3 times per week, by subcutaneous injection. We referred to KDOQI clinical practice guidelines and clinical practice recommendations for anemia in chronic kidney disease: [17] 2007 update of hemoglobin target and Clinical Practice Guidelines for Diagnosis and Treatment of renal anemia in China to determine the Hb target of this study between 110–130 g/L. Throughout the follow-up period, the patient’s Hb level, the rate of Hb change, the patient’s nutritional status, and iron metabolism were comprehensively evaluated to adjust the dose and interval of administration of ESAs or roxadustat. During the whole treatment of both groups, we timely supplemented iron when necessary (SF ≤ 100 ng/ml or TSAT ≤ 20%). In addition, we followed up with patients every month and recorded relevant laboratory test results, medication adherence, treatment costs, COVID-19 infection, co-morbidities, and clinical outcomes.

### Data collection

Basic patient information and laboratory test results were collected through an electronic medical record system at our hospital. We collected anemia related laboratory results, such as Hb and iron metabolism indicators. Lipid markers were collected such as total cholesterol, low-density lipoprotein (LDL), and high-density lipoprotein (HDL), cardiac markers such as left ventricular ejection fraction, brain natriuretic peptide (BNP), left ventricular end-systolic dimension (LVESD), and left ventricular end-diastolic dimension (LVEDD) were also collected. Patient complications, treatment costs, clinical outcomes, and other data were also collected and recorded through regular monthly visits. Additionally, we also supplemented the data from the electronic medical record system. Furthermore, We use Morisky Medical Adherence Scale-8 (MMAS-8) [18] to score medication adherence, with a maximum score of 8, indicating good adherence, 6–8 indicating moderate adherence, and<6 indicating poor adherence.

### Sample size calculation

Based on reading the literature [19] and our previous results, the standard deviation (SD) was 11.8 g/L in the roxadustat group and 14.7 g/L in the control group. The mean difference in ΔHb between the two groups was 2.78 g/L, the noninferiority margin was −4 g/L, α ＝ 0.05, power ＝ 0.8. We divided the treatment and control groups according to 1:4 and calculated the sample size using PASS software (Version 15). Considering potential dropouts, at least 30 patients were included in the roxadustat group and 118 in the ESAs group. This study included 34 patients in the roxadustat group and 120 patients in the ESAs group.

### Statistical analysis

The statistical analysis and plotting were performed using RStudio version 1.3 and Adobe Illustrator CS6. Measurement data that followed a normal distribution were presented as mean ± SD. For non-normally distributed data, the representation was Median [Min, Max]. Statistical analysis for normally distributed data was performed using the T-Test, and for non-normally distributed data, the Wilcoxon-Test was used. Categorical variables were expressed as a percentage (%), and statistical analysis for these variables was carried out using the Chi-square test or Fisher’s exact test. Intra-group differences were compared using a Single-factor repeated measure analysis of variance. The correlation between variables was analyzed using Pearson correlation analysis. A statistically significant difference was indicated by *P<0.05*.

## Results

### Baseline characteristics

A total of 154 patients were enrolled in this study, including 95 males and 59 females, with 34 in the roxadustat group and 120 in the ESAs group. The average age of patients in the two groups was 55.6 ± 16.1 years and 58.1 ± 3.4 years, respectively, and the average duration of PD was 34.1 ± 32.7 months and 34 ± 40.2 months, respectively. The primary causes of CKD in these patients were as follows: chronic glomerular nephritis (29.2%), diabetic nephropathy (29.9%), hypertensive nephropathy (20.8%) and others (20.1%). The mean baseline Hb level was 110 ± 9.98 g/L in the roxadustat group and 110 ±16.6 g/L in the ESAs group, which was not significantly different. The normal range of blood calcium in our center is 2.16–2.55mmol/L, and no hypercalcemia occurred in all included patients. We divided them into subgroups based on blood calcium levels of 2.16 mmol/L, and the results showed that there was no significant difference between the two groups of patients in the subgroups with blood calcium levels above and below 2.16 mmol/L (*P* = *0.323*, *P* = *0.707*). In addition, the iron related parameters of the two groups were also not significantly different. Basic information and clinical characteristics of the patients were not significantly different between the two groups (*P > 0.05*), refer to Table 1 for the baseline characteristics.

**Table 1:** Main baseline characteristics of patients included in the study.

Parameter	Roxadustat (N = 34)	ESAs (N = 120)	*P*-value
**Sex**			
Male	23 (67.6%)	72 (60.0%)	0.542
Female	11 (32.4%)	48 (40.0%)	
**Age (years)**	55.6 ± 16.1	58.1 ± 13.4	0.407
**Duration of dialysis (months)**	34.1 ± 32.7	34.0 ± 40.2	0.995
**Smoke**			
No	18 (52.9%)	81 (67.5%)	0.173
Yes	16 (47.1%)	39 (32.5%)	
**Etiology of CKD**			
Chronic glomerular nephritis	10 (29.4%)	35 (29.2%)	0.936
Diabetic nephropathy	9 (26.5%)	37 (30.8%)	
Hypertensive nephropathy	7 (20.6%)	25 (20.8%)	
Others	8 (23.5%)	23 (19.2%)	
**Weekly Kt/V**			
≥1.7	24 (70.6%)	76 (63.3%)	0.563
<1.7	10 (29.4%)	44 (36.7%)	
**CAPD schemes**			
1.5%x3 + 2.5%x1	25 (73.5%)	83 (69.2%)	0.781
1.5%x2 + 2.5%x2	9 (26.5%)	37 (30.8%)	
**Residence**			
City	15 (44.1%)	42 (35.0%)	0.441
Rural	19 (55.9%)	78 (65.0%)	
**Cardiac function indicators**			
BNP (pg/ml)	1160 ± 1200	1130 ± 1330	0.882
LVEDD (mm)	51.6 ± 2.39	51.5 ± 3.05	0.807
LVESD (mm)	35.7 ± 4.08	35.5 ± 4.09	0.825
LVEF (%)	49.9 ± 7.17	49.9 ± 5.67	0.963
**Iron metabolism indicators**			
Ferritin (ng/ml)	259 ± 252	266 ± 266	0.891
Serum iron (umol/L)	10.4 ± 5.75	12.4 ± 8.88	0.128
Transferrin saturation (%)	23.2 ± 4.01	23.8 ± 5.10	0.503
**Blood lipid indicators**			
Total cholesterol (mmol/L)	4.29 ± 0.779	4.62 ± 1.19	0.062
Triglyceride (mmol/L)	2.21 ± 1.49	1.98 ± 1.28	0.424
LDL-cholesterol (mmol/L)	3.71 ± 1.43	3.57 ± 1.46	0.613
HDL-cholesterol (mmol/L)	1.36 ± 0.558	1.39 ± 0.571	0.771
**Other laboratory indicators**			
Albumin (g/L)	36.2 ± 4.32)	37.1 ± 4.49	0.296
White blood cells (10^9^/L)	6.60 ± 1.96	6.51 ± 2.54	0.835
Red blood cell (10^12^/L)	3.15 ± 0.566	3.16 ± 0.691	0.881
Hemoglobin (g/L)	110 ± 9.98	110 ± 16.6	0.974
hs-CRP (mg/L)	26.2 ± 31.6	31.1 ± 46.6	0.482
Creatinine (umol/L)	706 ± 186	787 ± 399	0.094
Urea nitrogen (mmo/L)	27.4 ± 7.14	27.7 ± 9.04	0.798
Uric acid (umol/L)	401 ± 107	414 ± 128	0.564
Potassium (mmol/L)	4.38 ± 0.679	4.53 ± 0.722	0.256
Calcium (mmol/L)	2.03 ± 0.137	2.12 ± 0.210	**0.004**
Sodium (mmol/L)	139 ± 3.58	139 ± 4.18	0.483
Phosphorus (mmol/L)	1.49 ± 0.514	1.68 ± 0.558	0.075
iPTH (pg/ml)	369 ± 727	368 ± 362	0.997

ESAs, Erythropoiesis-stimulating agents; CKD, chronic kidney disease; Kt/V, urea clearance; CAPD, continuous ambulatory peritoneal dialysis; BNP, brain natriuretic peptide; hs-CRP, high-sensitivity C-reactive protein; LDL, Low-Density Lipoprotein; HDL, High-Density Lipoprotein; LVEDD, left ventricular end-diastolic dimension; LVESD, left ventricular end- systolic dimension; LVEF, left ventricular ejection fraction; iPTH, intact parathyroid hormone.

Continuous variables are expressed as mean ± standard deviation. Categorical variables are represented as n (%).

T-Test or Wilcoxon-Test was used for continuous variables, and Chi-Squared test or Fisher’s exact test was used for discontinuous variables.

### The effect of treating renal anemia

**Fig 2:**
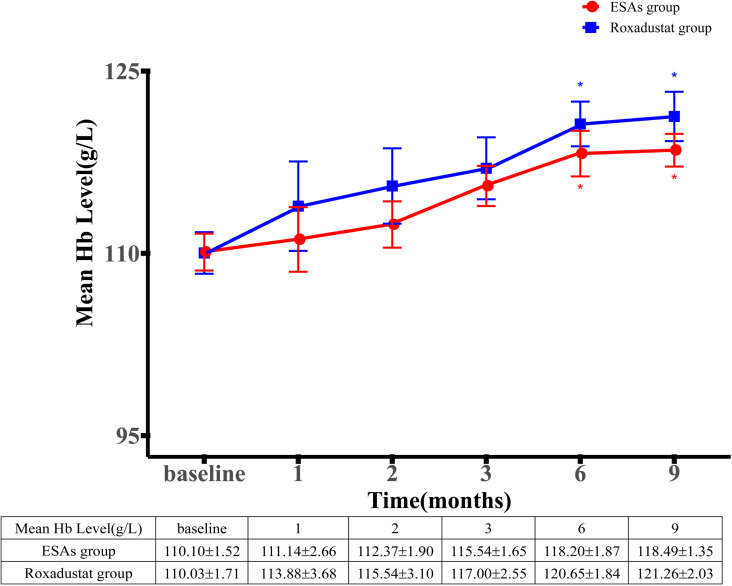
Mean hemoglobin levels of Roxadustat and ESAs groups over time. * *P* <  0.05 versus baseline level. Hb: hemoglobin; ESAs: erythropoiesis-stimulating agents.

The baseline values of Hb in the roxadustat and ESAs groups were 110.03 ± 1.71 g/L and 110.1 ± 1.52 g/L, respectively, with no significant difference between groups (*P = 0.974*), and we observed an increasing trend in Hb in both groups over time. In the roxadustat group, the mean Hb levels increased to 113.88 ± 3.68 g/L at month 1, 115.54 ± 3.1 g/L at month 2, 117 ± 2.55 g/L month 3, 120.65 ± 1.84 g/L month 6 and 121.26 ± 2.03 g/L at month 9, respectively, showing significant differences from baseline values at the 6th and 9th months (*P* = *0.046*, *P* = *0.029*). Similarly, in the ESAs group, the values of Hb were 111.14 ± 2.66 g/L at month 1, 112.37 ± 1.9 g/L at month 2, 115.54 ± 1.65 g/L month 3, 118.2 ± 1.87 g/L month 6 and 118.49 ± 1.35 g/L at month 9, respectively, and changed significantly from baseline at the 6th and 9th months (*P = 0.028*, *P = 0.02*) (Fig 2).

### Effects on cholesterol

**Fig 3:**
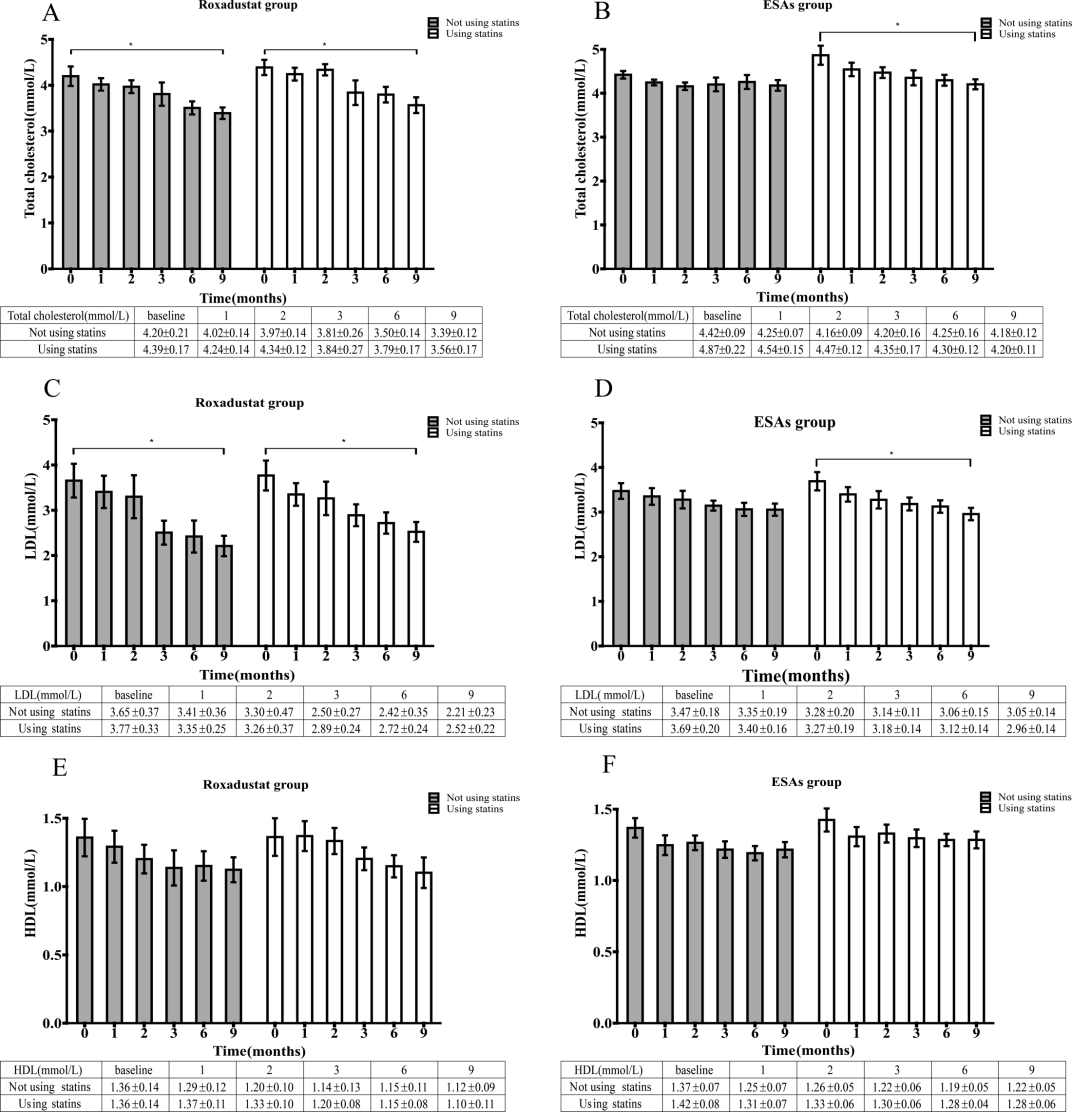
Mean total cholesterol levels over time in the roxadustat group (A) and ESAs group (B) according to the statins subgroup. Mean LDL-cholesterol levels over time in the roxadustat group (C) and ESAs group (D) according to the statins subgroup. Mean HDL-cholesterol levels over time in the roxadustat group (E) and ESAs group (F) according to the statins subgroup. * *P* <  0.05 versus the corresponding baseline level. LDL: Low-Density Lipoprotein; HDL: High-Density Lipoprotein; ESAs: erythropoiesis-stimulating agents.

We divided patients in the roxadustat and ESAs groups into statin and non-statin subgroups according to whether they received statins. And compared their changes in total cholesterol, LDL, and HDL. The results showed that in the roxadustat group, both the statin subgroup and the non-statin subgroup, the total cholesterol levels of patients significantly decreased from baseline at the 9th month (3.56 ± 0.17 vs. 4.39 ± 0.17 mmol/L and 3.39 ± 0.12 vs. 4.2 ± 0.21 mmol/L), with statistical significance (*P = 0.019*, and *P = 0.019*). The specific values at each time node are shown in Fig 3A. However, in the ESAs group, only the statin subgroup showed a significant decrease in total cholesterol levels compared with baseline at the 9th month (4.2 ± 0.11 vs. 4.87 ± 0.22 mmol/L, *P=0.031*), and the total cholesterol value at each time node was listed in detail in Fig 3B.

In addition, the LDL levels of the statin subgroup and non-statin subgroup in the roxadustat group also significantly decreased from baseline at the 9th month (2.52 ± 0.22 vs. 3.77 ± 0.33 mmol/L and 2.21 ± 0.23 vs. 3.65 ± 0.37 mmol/L), with a significant difference (*P = 0.026*, and *P = 0.049*), the specific values at each time node were shown in Fig 3C. Similarly, in the ESAs group, we only found a significant decrease in LDL values from baseline at the 9th month in the statin subgroup (2.96 ± 0.14 vs. 3.69 ± 0.2 mmol/L, *P* = *0.023*), as shown in Fig 3D. However, throughout the entire observation period, there was no significant change in HDL levels in either the subgroups of the roxadustat group or the ESAs group, as shown in Fig 3E and Fig 3F.

### Effects on cardiac function

**Fig 4:**
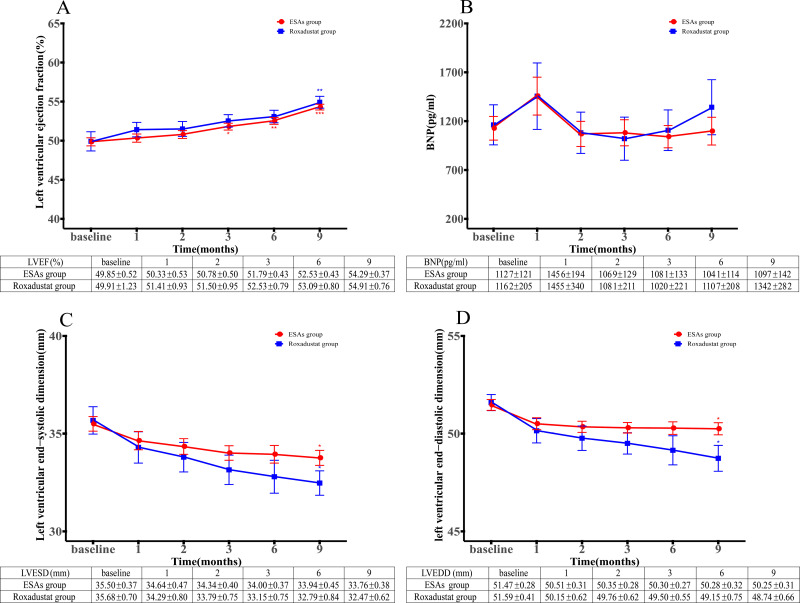
(A) Changes of left ventricular ejection fraction over time in Roxadustat and ESAs groups. (B) Changes of BNP over time in Roxadustat and ESAs groups. (C) Changes of LVESD over time in Roxadustat and ESAs groups. (D) Changes of LVEDD over time in Roxadustat and ESAs groups. * P < 0.05 versus the corresponding baseline level, **P < 0.01 versus the corresponding baseline level, ***P < 0.001 versus the corresponding baseline level. BNP: brain natriuretic peptide; LVESD: left ventricular end- systolic dimension; LVEDD: left ventricular end-diastolic dimension; ESAs: erythropoiesis-stimulating agents.

We conducted a statistical analysis of cardiac function-related indicators in the two groups. The primary indicators include: left ventricular ejection fraction, BNP, LVESD and LVEDD. The results indicated that the left ventricular ejection fraction in the roxadustat group was significantly higher than the baseline value at the 9th month (54.91 ± 0.76 vs. 49.91 ± 1.23%, *P = 0.002*), and the ESAs group was significantly higher than the baseline value at the 3rd, 6th and 9th months (51.79 ± 0.43% vs. 49.85 ± 0.52%, 52.53 ± 0.43% vs. 49.85 ± 0.52% and 54.29 ± 0.37% vs. 49.85 ± 0.52%), and the difference was statistically significant (*P = 0.039*, *P < 0.001* and *P < 0.001*), as shown in Fig 4A. During the whole follow-up period, BNP values in both groups did not change significantly, as shown in Fig 4B.

The LVESD of patients in both roxadustat group and the ESAs group showed a decreasing trend over time, and significantly changed from baseline value to the 9th month (32.47 ± 0.62 mm vs. 35.68 ± 0.7 mm and 33.76 ± 0.38 mm vs. 35.5 ± 0.37 mm), with statistical significance (*P = 0.033* and *P = 0.033*), as shown in Fig 4C. Similarly, the LVEDD values of the roxadustat group and ESAs group also showed a downward trend, and both decreased significantly from the baseline value to the 9th month (48.74 ± 0.66 mm vs. 51.59 ± 0.41 mm and 50.25 ± 0.31 vs. 51.47 ± 0.28 mm), with statistically significant differences (*P = 0.014* and *P = 0.043*), as shown in Fig 4D.

### Medication adherence

**Fig 5:**
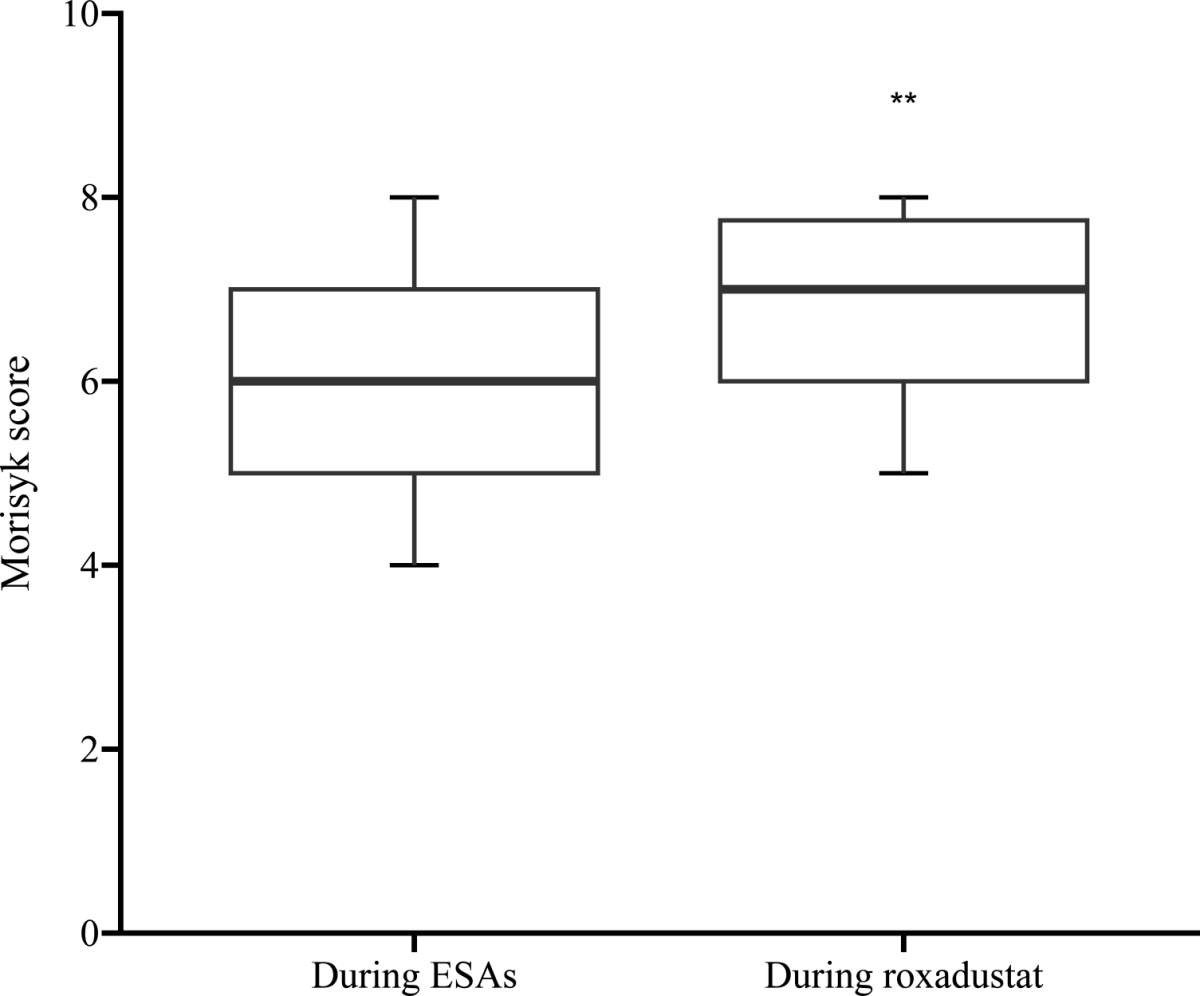
Morisky score for ESAs group and Roxadustat group. **P < 0.01 versus the ESAs group. ESAs: erythropoiesis-stimulating agents.

We compared medication adherence between two groups during the COVID-19 pandemic. The results showed that the Morisky score was 7 (5, 8) for the roxadustat group and 6 (4, 8) for the ESAs group, indicating a significant difference (*P<0.01*) (Fig 5). Specifically, 9 individuals in the roxadustat group and 5 in the ESAs group scored 8 points, 20 and 80 individuals scored 6–8 points, and 5 and 35 individuals scored less than 6 points in the respective groups.

### Drug expenditure

**Fig 6:**
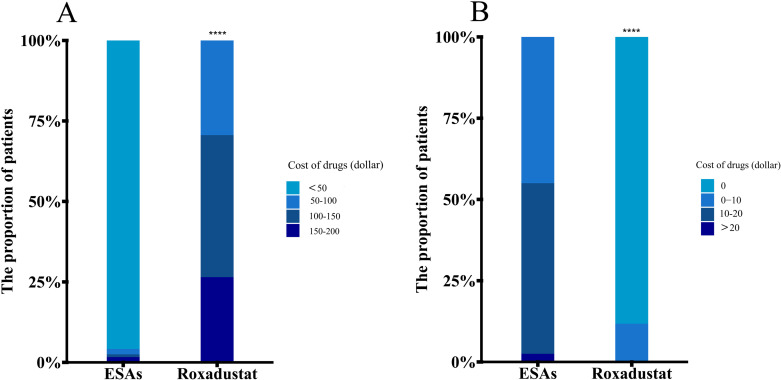
(A) Monthly drug costs of patients in the ESAs group and Roxadustat group. (B) Additional monthly costs of measures to correct renal anemia in addition to ESAs and roxadustat. ****P < 0.0001 versus the ESAs group.

In our study, we compared the monthly drug costs of patients using roxadustat and ESAs. The results indicated that 95.83% of patients in the ESAs group spent less than 50 dollars per month, while 29.41% of patients in the roxadustat group spent 50–100 dollars per month, 44.12% spent 100–150 dollars per month, and 26.47% spent 150–200 dollars per month. There was a significant difference between the two groups (*P < 0.001*), as shown in Fig 6A. Additionally, we also compared the monthly costs of additional measures to correct renal anemia, such as iron use and blood transfusions, among the two groups. The results revealed that 45% of patients in the ESAs group incurred an extra cost of 0–10 dollars per month, 52.5% spent an extra 10–20 dollars per month, and 2.5% spent more than 20 dollars per month. However, in the roxadustat group, 88.24% of patients had no additional monthly costs, and 11.76% had an extra cost of 0–10 dollars per month. Again, there was a significant difference between the two groups (*P < 0.001*), as shown in Fig 6B.

### Clinical outcome

We conducted a comparative analysis of the clinical outcomes of patients in the two groups after 9 months of follow-up, and the results showed that the number of patients infected with COVID-19 in the roxadustat group and ESAs group was 24 and 105, respectively, with a significant difference (*P = 0.036*). During the follow-up period, the number of hospitalized patients in the roxadustat group and ESAs group was 11 and 65, respectively, and the number of deaths due to COVID-19 infection was 3 and 33, respectively, both with significant differences (*P = 0.04* and *P = 0.041*). We also performed a statistical analysis of ICU admission, respiratory support, and overall mortality in both groups, as shown in Table 2.

**Table 2:** Summary of clinical outcomes after 9 months.

Parameter	Roxadustat (N = 34)	ESAs (N = 120)	*P*-value
**COVID-19**			
No	10 (29.4%)	15 (12.5%)	0.036
Yes	24 (70.6%)	105 (87.5%)	
**Hospitalization treatment**			
No	23 (67.6%)	55 (45.8%)	0.040
Yes	11 (32.4%)	65 (54.2%)	
**ICU treatment**			
No	32 (94.1%)	93 (77.5%)	0.053
Yes	2 (5.9%)	27 (22.5%)	
**Respiratory support therapy**			
No	31 (91.2%)	89 (74.2%)	0.061
Yes	3 (8.8%)	31 (25.8%)	
**All-cause mortality**			
No	28 (82.4%)	78 (65.0%)	0.086
Yes	6 (17.6%)	42 (35.0%)	
**Mortality caused by COVID-19**			
No	31 (91.2%)	87 (72.5%)	0.041
Yes	3 (8.8%)	33 (27.5%)	

ESAs, Erythropoiesis-stimulating agents; ICU, Intensive Care Unit.

Categorical variables are represented as n (%).

The Chi-Square Test or Fisher’s exact test was used for discontinuous variables.

## Discussion

Patients undergoing PD are less likely to contract COVID-19 compared to those undergoing HD. Primarily because PD can be performed at home, whereas HD patients must visit dialysis centers regularly to complete the therapy, which increases their risk of exposure to COVID-19 [20]. However, renal anemia, as a common complication of CKD, requires regular treatment and follow-up even in the context of the COVID-19 pandemic, so it is particularly important to choose safe and effective treatment options. However, studies related to the management of renal anemia during the pandemic are limited and far from being clarified.

To address this gap, we conducted this research, which is expected to bring new ideas for how to better manage renal anemia during the later pandemic. Roxadustat and ESAs are currently common drugs for the clinical treatment of renal anemia, and their modes of administration and drug costs are different. COVID-19 can cause damage to multiple organ systems, not just the respiratory system. However, anemia can also lead to worse clinical outcomes and higher mortality in patients with respiratory diseases [21,22], indicating that anemia and COVID-19 are interrelated. Roxadustat and ESAs can correct anemia by different mechanisms, the former mimics the body’s natural response to hypoxia, while the latter acts directly on red blood cell progenitors to improve anemia. Although there are fewer studies on the effectiveness of roxadustat in PD patients compared with HD, previous studies have shown that roxadustat can improve renal anemia in PD patients [23,24]. Our results showed that both roxadustat and ESAs were able to maintain Hb in a relatively stable state throughout the follow-up period, with the mean Hb in both groups above 110 g/L at all time points. Due to the concentrated outbreak of COVID-19 in China from December 7, 2022, to January 31, 2023, our statistical results indicated that the Hb of patients in both groups remained relatively stable during the first three months of follow-up. A significant increase was not observed until the sixth month. The stability in the first three months could be attributed to several factors, the overall condition of most patients became worse due to the infection of COVID-19, and in the sixth month after the physical condition of patients improved, drugs could better play their role in correcting anemia.

The prevalence of abnormal lipid metabolism in CKD patients is higher than in the general population, and hyperlipidemia is the main disease [25]. However, studies have shown that after removing the effects of inflammation and malnutrition, blood lipids are positively correlated with all-cause mortality and cardiovascular mortality in dialysis patients [26], indicating that active control of blood lipid levels is of great significance. Our results suggest that roxadustat has a cholesterol-lowering and LDL-lowering effect in PD patients compared with ESAs, which is intended to reduce the incidence of cardiovascular events in patients. The HIF pathway can improve lipid metabolism through several aspects, which may mainly involve insulin-induced gene 2, sterol regulatory element-binding protein 1C, and alkaline ceramidase2 [27,28].

Previous studies have demonstrated that controlling Hb to the target is beneficial for reducing left ventricular mass index and stabilizing BNP [29,30]. Our results showed that with the improvement of renal anemia in both groups, the left ventricular ejection fraction in both groups was increased, LVSED and LVEDD were decreased, and BNP was relatively stable. These improvements were primarily because the myocardial load level of CKD patients was reduced after the correction of the Hb level. Additionally, we observed that the roxadustat group showed a more significant increase in left ventricular ejection fraction and decreased LVESD and LVEDD levels than the ESAs group. This is likely because roxadustat has a lipid-lowering effect that benefits cardiac function more than ESAs.

Our results indicated that patients in the roxadustat group had significantly higher medication adherence than those in the ESAs group. This is partly because roxadustat is administered orally, making it more convenient and less painful than subcutaneous injections of ESAs. Additionally, during the COVID-19 pandemic, patients in the ESAs group had to visit clinics or hospitals for their injections, which likely contributed to reduced medication adherence. Although the monthly cost of medication was lower in the ESAs group than in the roxadustat group, the cost of other adjuvant treatments to correct renal anemia was significantly higher in this group than in the roxadustat group. This may be due on the one hand to the fact that most patients were infected with COVID-19 to varying degrees and showed varying degrees of inflammation in their bodies. However, previous studies have shown that the effect of roxadustat on improving anemia is not affected by inflammation [23]. Furthermore, it has been shown that roxadustat is involved in the processes of iron metabolism, such as iron absorption, transport, utilization, and recycling, in addition, this drug can also inhibit hepcidin [19,23,31].

Concerning the clinical outcome of COVID-19, our research results showed that the infection rate of COVID-19, the hospitalization rate, and the mortality rate caused by COVID-19 infection in the roxadustat group were lower than those in the ESAs group. This could be related to the different administration methods of the two drugs, as the ESAs group required patients to go to a clinic or hospital for subcutaneous injection, thereby increasing the risk of infection. Going through reading the literature, we learned that roxadustat may have a potential therapeutic effect on COVID-19, which may also be the reason for the better clinical outcome of patients in the roxadustat group [32]. In addition, some studies link the severity of COVID-19 to hyperferritinemia [33] and thus, to ferroptosis [34]. Interestingly, drugs belonging to the HIF-PHI class are potent inhibitors of ferroptosis working via the inhibition of HIF prolyl hydroxylases [35]. Therefore, roxadustat may bring additional benefits to the clinical outcomes of COVID-19 compared to ESAs.

Overall, during the pandemic of COVID-19, both roxadustat and ESAs were effective in improving the renal anemia status of patients undergoing PD. However, patients in the roxadustat group demonstrated better medication compliance. It is important to note that roxadustat is administered orally and ESAs are administered by injections. This fundamental difference may influence patients’ adherence to their medication regimens and, in turn, impact other clinical outcome indicators. While the overall drug cost of the roxadustat group was higher, the additional cost for assisting in correcting anemia was lower. In terms of clinical outcomes related to COVID-19, the roxadustat group was superior to the ESAs group. We look forward to more multi-center prospective studies in the future on the treatment of anemia in PD patients in the context of viral pandemics, including details such as the availability and cost of drugs such as roxadustat or ESAs.

### Limitation

This was a single-center retrospective study with a relatively small number of cases and a relatively short follow-up time. At present, COVID-19 has shown a trend of globalization and has lasted for several years. People’s attention to it has gradually decreased. However, infection with COVID-19 will have a long-term and chronic impact on the human body. Studying the treatment plan for renal anemia in patients undergoing dialysis therapy during the pandemic is conducive to providing a reference for the pandemic of other viruses. There was a difference in sample size between the two groups in this study (34 in the roxadustat group and 120 in the ESAs group), which may introduce bias. We ensure that the collected samples came from the same population, and we have increased the baseline data of patients as much as possible and classified them in detail to ensure comparability between the two groups. In addition, there is a possibility of confounding or bias due to unrecognized variables, although we have adjusted the baseline characteristic variables between the two groups whenever possible.

## Key message

1. During COVID-19, both roxadustat and ESAs could effectively improve renal anemia in PD patients.
2. The medication compliance of roxadustat was better than that of ESAs.
3. The drug cost of roxadustat was higher, but the cost of auxiliary treatment measures for anemia correction was less than that of ESAs.
4. The clinical outcome of PD patients was better in roxadustat group than in ESAs group.

## Supporting information

S1 Checklist CONSORT 2010 checklist of information to include when reporting a randomised trial * . (DOC)

S1 File Approval by the research ethics committee. (JPG)

S1 Data Study data. (XLSX)
