# Supplementary figures and images for: Roxadustat effectiveness versus ESAs in peritoneal dialysis patients during the COVID-19 pandemic: A retrospective study
Source: PLoS One. 2025 Mar 26;20(3):e0320536. doi: 10.1371/journal.pone.0320536 (PMC11940824; doi:10.1371/journal.pone.0320536)

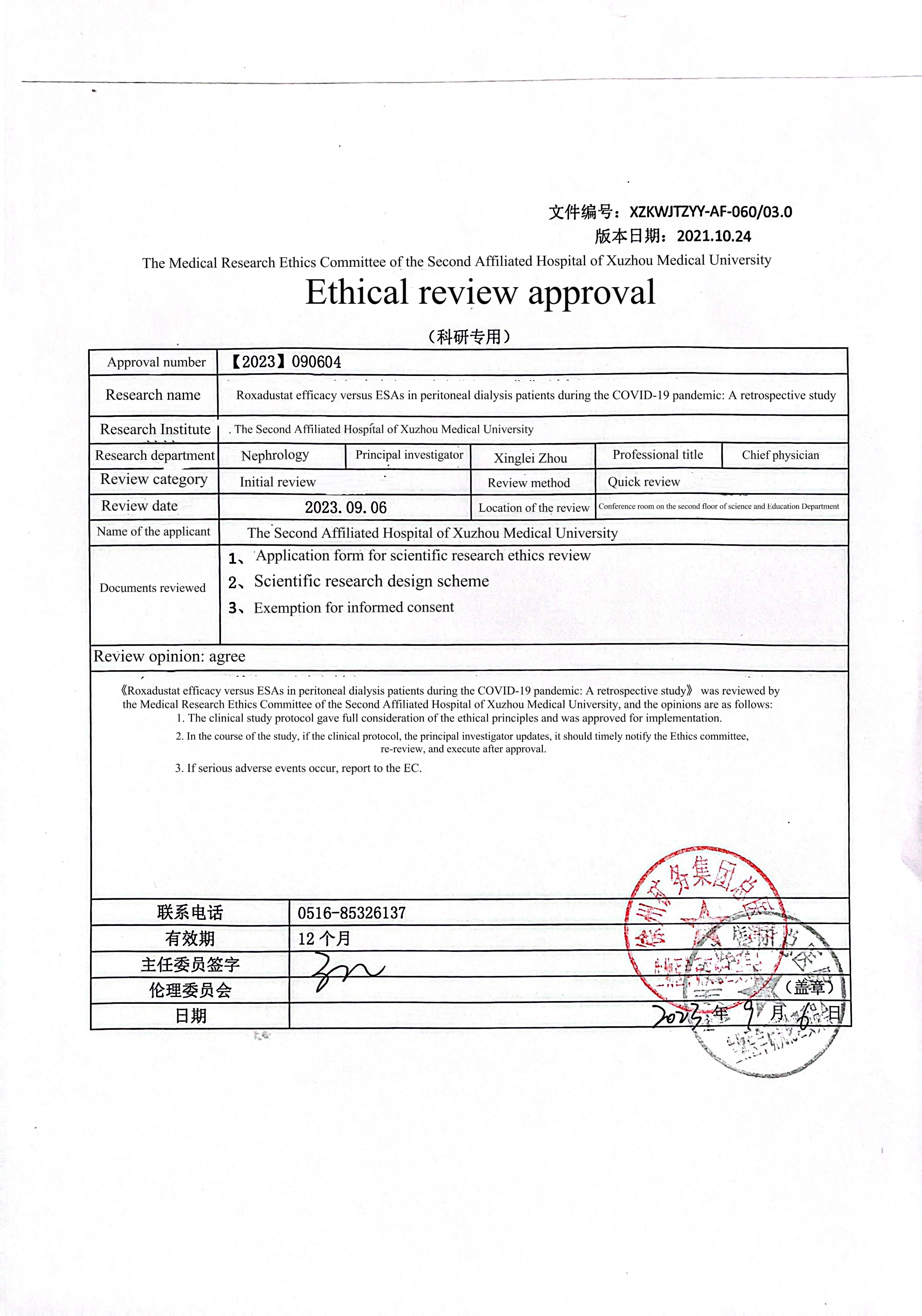

Supplement: S1 File — (JPG) [file pone.0320536.s002.jpg]
